# Engineered VP2-NC antigen expressed in an NDV vector elicits enhanced protection against the challenge of the novel variant infectious bursal disease virus

**DOI:** 10.1016/j.psj.2026.107090

**Published:** 2026-05-08

**Authors:** Ziyan Zhang, Changhai Liu, Yaodong Zhang, Jun Dai, Lei Tan, Yingjie Sun, Cuiping Song, Yang Qu, Ning Tang, Ying Liao, Chan Ding, Xusheng Qiu

**Affiliations:** a Shanghai Veterinary Research Institute, Chinese Academy of Agricultural Sciences, Shanghai 200241, PR China; b Experimental Animal Center, Zunyi Medical University, Zunyi 563000, China

**Keywords:** NDV, nVarIBDV, Bivalent vaccine, Virus assembly

## Abstract

To enhance the efficacy of Newcastle disease virus (NDV)-vectored bivalent vaccines against novel variant infectious bursal disease virus (nVarIBDV), we engineered recombinant NDV strains expressing nVarIBDV VP2 proteins fused with the viral nucleocapsid NC motif, thereby leveraging the NDV assembly pathway. The resulting vaccine candidates, rDM-VP2NC and rDM-VP2mNC, exhibited markedly enhanced VP2 expression in vitro. Furthermore, the NC motif promoted VP2 co‑localization with the NDV M protein near the cell membrane and facilitated its efficient incorporation into budding virus-like particles (VLPs) and virions. In chickens challenged with a nVarIBDV strain, immunization with rDM‑VP2NC or rDM‑VP2mNC provided superior protection compared to the parental rDM‑VP2 strain, as demonstrated by a drastic reduction in bursal viral load and the absence of detectable pathological lesions. These results establish the NC motif as a key determinant for enhancing antigen assembly and immunogenicity in NDV vectors. Collectively, this work presents a novel strategy for engineering NDV as a vector that directly packages protein cargo within virions, with broad implications for vaccine and oncolytic virotherapy development.

## Highlights

• The NC motif promotes a foreign protein binding to NDV M protein and incorporation into virions.
• Fusion of NC motif significantly boosts nVarIBDV VP2 protein levels in recombinant NDV vectors.
• Vaccines expressing NC-fused VP2 provide enhanced protection against nVarIBDV, with reduced viral load and pathology.
• This work presents a strategy to engineer NDV vectors that directly package protein cargo, improving vaccine efficacy.

## Introduction

Infectious bursal disease (IBD) is an acute and highly contagious disease caused by infectious bursal disease virus (IBDV). The virus selectively replicates within the developing B lymphocytes, which ultimately results in the necrosis and disintegration of antibody-producing B cell precursors in the bursa of Fabricius. This process leads to severe immunosuppression, thereby enhancing the susceptibility of chickens aged between 3 and 6 weeks to other pathogens (Zhang et al., 2022). Vaccination serves as the principal method for controlling IBD, with both live-attenuated and inactivated vaccines being widely adopted in current veterinary practice. However, the ongoing emergence of new IBDV variants is gradually weakening the protective immunity offered by these commercial vaccines.

There are two serotypes of IBDV (Serotypes I and II), and only serotype I strains are pathogenic to chickens (Bo et al., 2023; Mahgoub et al., 2012). According to their pathogenicity and antigenicity, the serotype I IBDV strains can be further divided into classical strains(cIBDV) (Cosgrove, 1962), very virulent strains (vvIBDV) (Chettle et al., 1989), variant strains (varIBDV) (Jackwood and Saif, 1987), and novel variant strains (nVarIBDV) (Fan et al., 2019b; Legnardi et al., 2023). Since the genome of IBDV consists of two segments, A and B, a recently proposed improved scheme for classifying the genotypes of IBDV takes into account both VP1 (encoded by segment B) and VP2 (encoded by segment A) (Wang et al., 2021a). In this novel classification scheme, the cIBDV, vvIBDV, VarIBDV, and nVarIBDV correspond to genotypes A1B1, A3B2, A2B1 (including A2aB1, A2bB1, and A2cB1), and A2dB1, respectively (Mosad et al., 2024; Wang et al., 2021a; Zhang et al., 2022).

The nVarIBDV (A2dB1) is a type of novel variant strains that has recently appeared in several countries, including China (Fan et al., 2019b; Huang et al., 2022), Japan (Myint et al., 2021; Takahashi et al., 2024), South Korea (Thai et al., 2021), Egypt (Legnardi et al., 2023), and Malaysia (Aliyu et al., 2021). These variant strains do not share the same phylogenetic lineage with the varIBDV strains isolated in the United States in 1985 (Thai et al., 2021; Wang et al., 2021a). Unlike classical strains, the nVarIBDV does not lead to death or clinical symptoms in chickens; however, severe atrophy of the bursa of Fabricius, an important immune organs of chickens, can be observed even in the presence of antibodies induced by commercial IBDV vaccines (Aliyu et al., 2024; Fan et al., 2019b, 2020; Huang et al., 2022). The prevalence of nVarIBDV will weaken the immune effect of commercial vaccines and further affect the quality of poultry eggs, undoubtedly posing security and economic risks to the poultry industry.

The VP2 protein is the major capsid protein of IBDV, which contains key neutralizing epitopes (Azad et al., 1991; Becht et al., 1988; Heine and Boyle, 1993) and is widely used as the immunogenic antigen in the development of recombinant virus-vectored vaccines (Boursnell et al., 1990; Dey et al., 2017; Huang et al., 2004; Liu et al., 2024; Okura et al., 2021; Shah et al., 2022). Newcastle Disease Virus (NDV) is a promising vector for the IBDV VP2 protein, since the NDV is a mature vaccine vector and has been widely utilized in various vectored vaccine researches (Ge et al., 2011; Hu et al., 2020; Viktorova et al., 2018). It is well known that NDV vaccines exhibit strong immunogenicity, eliciting robust humoral and cellular immune responses, along with strong mucosal immunity in the digestive tract, making it an ideal vector for developing IBDV vaccines.

Previous studies have successfully generated recombinant NDV strains stably expressing IBDV VP2 proteins (Dey et al., 2017; Huang et al., 2004; Liu et al., 2024), demonstrating dual protection against both IBDV and NDV in specific-pathogen-free (SPF) chickens. To advance these NDV-vectored bivalent vaccines, strategic optimization of VP2 expression and presentation is essential to enhance their immunogenic efficacy. Enhancing immunogenicity through exogenous protein incorporation into viral particles is a proven strategy, exemplified by NDV-vectored vaccines for influenza, coronavirus and so on (Liu et al., 2018; Sun et al., 2020; Wu et al., 2019). This typically involves fusing target antigens with the cytoplasmic region of NDV glycoproteins, exploiting their M protein interactions for virion packaging (Adu-Gyamfi et al., 2016; El Najjar et al., 2014; Pantua et al., 2006). However, IBDV VP2—a soluble structural protein lacking membrane-targeting domains—poses a unique challenge, demanding alternative approaches for efficient NDV virion incorporation.

The M protein of paramyxoviruses plays a critical role in virion assembly, integrating not only the surface glycoproteins HN and F but also the NP into the viral envelope (Battisti et al., 2012; El Najjar et al., 2014; Pantua et al., 2006; Ray et al., 2016). In light of this, we designed a recombinant NDV to stably express the nVarIBDV VP2 protein by fusing it to the C-terminal motif of the NDV NP protein (NC), which interacts directly with M (Ray et al., 2016). This study evaluates the expression, immunogenicity, and protective efficacy of this NC-VP2 construct against nVarIBDV challenge. Our approach establishes a novel paradigm for optimizing exogenous protein expression in viral vectors, offering innovative insights for vaccine development.

## Materials and methods

### Cell lines and viruses

The cell lines HEK-293T, HeLa, and DF-1 (a chicken fibroblast line) were obtained from the American Type Culture Collection (ATCC) and preserved in our lab. Cells were cultured in either Dulbecco's modified Eagle's medium (DMEM) or RPMI-1640 medium (both from Gibco, Grand Island, NY, USA), each supplemented with 10 % fetal bovine serum (FBS, Gibco), and incubated at 37°C with 5 % CO₂.

The nVarIBDV strain SD-LY-CN-2020 was isolated, identified and preserved in our lab (Zhang et al., 2026). Its full-length genome sequences are available in GenBank under accession numbers OM307063 (segment A) and OM307064 (segment B). The NDV strain LaSota/46 was obtained from the China Institute of Veterinary Drug Control (Beijing, China), and the DM strain was derived from it via reverse genetics previously in our lab (Peng et al., 2022). SPF chicken embryos for preparation of viruses and SPF chickens were from Beijing Boehringer Ingelheim Viton Biotechnology Co., Ltd. (Beijing, China). The NDV virus was propagated in 10-day-old SPF embryonated chicken eggs, with infectious allantoic fluid collected 24 to 120 hours after inoculation, as described previously (Gough et al., 1988).

### Plasmids construction and mutagenesis

The open reading frames (ORFs) of green fluorescent protein (GFP) and VP2 protein were ligated into the pCMV-HA vector using the Mut Express® II Fast Mutagenesis Kit V2 (China), yielding the constructs pCMV-HA-GFP and pCMV-HA-VP2. Subsequently, the nucleotide sequences corresponding to the NC90 (90 bp), NC60 (60 bp), and NC30 (30 bp) motifs from the NDV LaSota NP gene were individually amplified and fused in-frame to the C-terminus of either GFP or the HA ORF. These recombinant plasmids were designated as pCMV-HA-GFP-NC90, -NC60, -NC30, and pCMV-HA-VP2NC to express recombinant HA-GFPNC90, HA-GFPNC60, HA-GFPNC30, and HA-VP2NC proteins. To examine the functional contribution of individual residues within the NDV NC30 motif, we introduced single alanine substitutions at each of ten consecutive amino acid positions (480–489) in the NC30 motif of the pCMV-HA-GFP-NC30 plasmid. The resulting mutant plasmids were termed pCMV-HA-GFPNC-S480A, -Q481A, -D482A, -N483A, -D484A, -T485A, -D486A, -W487A, -G488A, and -Y489A.

### Immunofluorescence

The immunofluorescence assay (IFA) was performed on transfected 293T and HeLa cells as previously described (Sun et al., 2019). Briefly, at approximately 70 % confluence, 293T cells were transfected using PEI (Thermo Fisher Scientific/Polysciences, Inc.), and HeLa cells were transfected using Lipofectamine 2000 (Thermo Fisher Scientific). At 24, 36, or 48 h post-transfection (hpt), the cells were fixed with 4 % paraformaldehyde at room temperature for 15 min, permeabilized with 0.5 % Triton X-100 for 10 min, and blocked with 3 % bovine serum albumin (BSA) for 1 h at 37 °C. Samples were then incubated with primary antibodies (e.g., anti-FLAG, MBL, or anti-HA, ABclonal) for 3 h at 37 °C, followed by incubation with fluorescent secondary antibodies (Invitrogen) for 1 h at 37 °C. After washing with Tris-buffered saline with Tween 20 (TBST), the cells on coverslips were mounted using an anti-fade medium containing DAPI (Yeason). Image acquisition was performed using a Zeiss LSM880 confocal microscope, and the images were analyzed using ImageJ software.

### Co-Immunoprecipitation (Co-IP) Assay

293T cells, maintained in DMEM supplemented with 10 % fetal bovine serum at 37°C, were seeded into 6-well plates and transfected with the relevant plasmids using PEI at a 1:3 (w/v) plasmid-to-PEI ratio. Cells were harvested and lysed at 18 hpt. For each well, 15 µL of anti-FLAG magnetic beads were prepared and equilibrated with TBST. Cell lysates were cleared by centrifugation at 12,000 rpm for 10 min at 4°C, and 48 µL of supernatant was aliquoted as the Input sample. The remaining lysate was incubated with the equilibrated beads overnight at 4°C with rotation. Following incubation, the beads were washed three times with TBST, and bound proteins were eluted by resuspension in 48 µL of TBST plus 12 µL of 5 × SDS loading buffer, followed by boiling at 100°C for 10 min. Precipitated proteins and Input controls were subsequently analyzed by Western blotting as previously described (Li et al., 2018).

### Virus-like particle (VLP) assay

The VLP assay was performed in 293T cells as previously described (Peng et al., 2022). Cells were seeded in 6-well plates and transfected at 80–90 % confluency with 2 µg per well of FLAG-M together with plasmids expressing GFP, VP2, or their derived mutants. All experiments were conducted in triplicate. At 48 hpt, the culture supernatant was collected and clarified by centrifugation at 5,000 × g for 10 min. The supernatant was then concentrated using an Amicon® Ultra 100 K device (Merck), and the final volume was adjusted to 100 µL with PBS. In parallel, cells were harvested in 1 mL of lysis buffer, and the lysate was cleared by centrifugation at 5,000 × g for 10 min to remove nuclei and debris. Both the concentrated supernatant (VLP fraction) and the cleared cell lysate were mixed with 2 × SDS-PAGE loading buffer, boiled at 100 °C for 10 min, and analyzed by Western blotting as previously described (Peng et al., 2022).

### Structure modeling

The deduced amino acid sequences of the M protein from the NDV LaSota strain and the VP2 protein from the nVarIBDV strain SD-LY-CN-2020 were submitted to the modeling D-I-TASSER server (https://aideepmed.com/D-I-TASSER/) for structural prediction (Zheng et al., 2025), and their three-dimensional models were subsequently generated. The interaction between the NDV NP protein and the NC motif was analyzed using the ZDOCK server (https://zdock.wenglab.org/) (Mintseris et al., 2007; Pierce et al., 2011).

### Recovery of recombinant NDVs (rNDVs)

The full-length plasmid pLaSota-rDM-wt, which carries the genomic cDNA of the NDV vaccine strain LaSota with R247K and S263R mutations in its M protein, was previously constructed based on the vector pTVT7R (Peng et al., 2022). The ORFs encoding VP2, VP2NC, and VP2mNC proteins, each flanked by NDV gene-end (GE) and gene-start (GS) motifs, were inserted into this plasmid between the P and M genes. Specifically, VP2NC90 uses the original codon sequence of the NC motif from the LaSota strain, whereas VP2mNC90 employs a more common codon sequence. The resulting recombinant full-length plasmids were designated as pTVT-rDM-VP2, pTVT-rDM-VP2NC, and pTVT-rDM-VP2mNC.

Recombinant NDVs expressing the foreign VP2, VP2NC, or VP2mNC proteins were rescued using our established methods (Peng et al., 2022). Briefly, BSR T7/5 cells were co-transfected with 5 µg of a full-length plasmid, 2 µg of pCI-NP, 2 µg of pCI-P, and 1 µg of pCI-L using Lipofectamine 2000 (Invitrogen). Following a 60-hour incubation at 37°C with 5 % CO₂ in DMEM medium containing 1 % FBS and 2 % pancreatic enzyme, the supernatant was harvested and inoculated into the allantoic cavities of 9-day-old SPF embryonated chicken eggs. Four days after inoculation, the allantoic fluid was collected and titrated by the hemagglutination (HA) assay. The rescued virus was then passaged through successive generations (up to 5 times) in SPF embryonated eggs for further study.

### Biological characterization of the generated virus and in vitro growth kinetics

The pathogenicity of the rNDV strains, including rDM-wt, -VP2, -VP2NC, and -VP2mNC, was evaluated through MDT and ICPI assays following WOAH protocols. The replication kinetics of those strains were analyzed in DF1 cells. Briefly, cells were plated at 1 × 10⁶ cells/well in 6-well plates, cultured in DMEM supplemented with 10 % FBS, and infected at a multiplicity of infection (MOI) of 5. Virus titers in the supernatants, harvested at 6-hour intervals from 6 to 36 h post‑infection (hpi), were determined via SYBR Green fluorescence quantitative PCR (RT-qPCR) or a 50 % tissue culture infective dose (TCID₅₀) assay as previously described (Zhang et al., 2025). Growth curves were then generated based on these titer measurements.

### Transmission electron microscopy (TEM)

Allantoic fluid containing each of the recombinant viruses was concentrated at 30,000 rpm for 3 h and purified by sucrose gradient centrifugation (Granzow et al., 1999). Subsequently, purified virus particles were subjected to transmission electron microscopy (TEM) observation.

DF-1 cells were infected with the recombinant viruses (rDM‑wt, rDM‑VP2, rDM‑VP2NC, and rDM‑VP2mNC) at an MOI of 5. At 18 hpi, the infected cells were fixed using immunoelectron microscopy specific fixative according to the protocol provided by Servicebio Technology Co., Ltd. (China) and then examined by TEM for morphological observation.

### Evaluation of the immunogenicity and protective efficacy of the rNDV in chickens

To evaluate the immunogenicity of the recombinant NDV strains, fifty 1-day-old SPF chicks were randomly assigned to five groups (n = 10 per group): a positive control group (PC), three vaccinated groups (G1, G2, G3), and an unvaccinated negative control group (NgC). Each group was housed in a separate isolator under controlled environmental conditions (28–30°C; 12 h light/dark cycle) with *ad libitum* access to food and water. At 7 days of age, chicks in groups PC, G1, G2, and G3 were vaccinated via the intraocular and intranasal routes with 10^7^ EID₅₀ of the corresponding rNDV strain (rDM-wt, -VP2, -VP2NC, or -VP2mNC, respectively) in 0.1 mL of PBS. Twenty-one days post-vaccination, birds in these groups were challenged with 50 BAD₅₀ (50 % Bursae Atrophy Dose) of the nVarIBDV strain SD-LY-CN-2020 (equivalent to approximately 6 × 10^6^ viral RNA copies) via the intraocular-nasal route, with reference to previous reports (Fan et al., 2019a, 2020; Wang et al., 2020). Serum samples were collected at 7 and 14 days post-vaccination and at 7 dpc (days post-challenge) for the detection of NDV- and IBDV-specific antibodies.

At 7 dpc, all birds were humanely euthanized by CO₂ asphyxiation for necropsy. The bursae of Fabricius and spleens were dissected, weighed, and photographed. Bursal atrophy was determined according to conventional standards, where a bursa‑to‑body weight index (BBIX) value of less than 0.7 was considered indicative of bursal atrophy (Fan et al., 2020; Lucio and Hitchner, 1979). Tissue samples were fixed in 10 % neutral buffered formalin and submitted to Wuhan Servicebio Company for paraffin embedding, sectioning, and hematoxylin and eosin (HE) staining for histopathological analysis. For viral load quantification, total RNA was extracted from bursal tissue using a commercial kit (Vazyme Biotech Co., Ltd., China). cDNA was synthesized from the extracted RNA using random hexamer primers (TransGen Biotech), and the viral RNA load of IBDV was subsequently quantified by RT-qPCR as previously described (Peng et al., 2022; Wang et al., 2009; Zhang et al., 2025). The relative expression levels of immune-related genes, including *IL-2, BLB2, IFN-α, IL-10, TNF-α*, and *IFN-γ*, were quantified by RT‑qPCR using GAPDH as the housekeeping gene, with specific primer pairs (Table 1) and protocols following a previous report (Gao et al., 2012; Xiao et al., 2025).

**Table 1:** The RT-qPCR primers used in this study.

Gene	Sequence(5′−3′)	GenBank
IL-2	F: GCTAATGACTACAGCTTATGGAGCA	AF000631.1
	R: TGGGTCTCAGTTGGTGTGTAGAG	
IFN-α	F: GACAGCCAACGCCAAAGC	GU119896.1
	R: GTCGCTGCTGTCCAAGCATT	
IL-10	F: AGCAGATCAAGGAGACGTTC	NM_001004414.2
	R: ATCAGCAGGTACTCCTCGAT	
TNF-α	F: GCTGTTCTATGACCGCCCAGTT	NM_204267.1
	R: AACAACCAGCTATGCACCCCA	
IFN-γ	F: CCTCCAACACCTCTTCAACATG	X92479
	R: TGGCGTGCGGTCAAT	
B2-BLB2	F: CCCTCGGCGTTCTTCTTCTG	
	R: ACTAACTGCTGCCGGTTGTAG	
GAPDH	F: GAACATCATCCCAGCGTCCA	NM_204305.1
	R: CGGCAGGTCAGGTCAACAAC	
IBDV	F: GAGCCTTCTGATGCCAACAAC	
	R: CAAATTGTAGGTCGAGGTCTCTGA	

### Statistical analysis

The Grayscale scanning of WB, viral HA titers, viral load of organs were statistically analyzed. The P values were generated by one-way ANOVA for multiple comparisons between groups.

### Data availability statements

The datasets generated during and/or analyzed in this study are available from the corresponding authors on reasonable request.

### Materials availability

Upon request, further information, resources, and reagents are available from the corresponding authors.

### Ethics statements

All animal experiments were approved by the Institutional Animal Care and Use Committee of the Shanghai Veterinary Research Institute (SHVRI), Chinese Academy of Agricultural Sciences (Project Application Number is SVLAC/XM-A-Y22032), and conducted in strict compliance with the committee’s guidelines and relevant ethical standards for animal welfare.

## Results

### The NDV NP tail promotes the binding of GFP and NDV M proteins

**Fig. 1:**
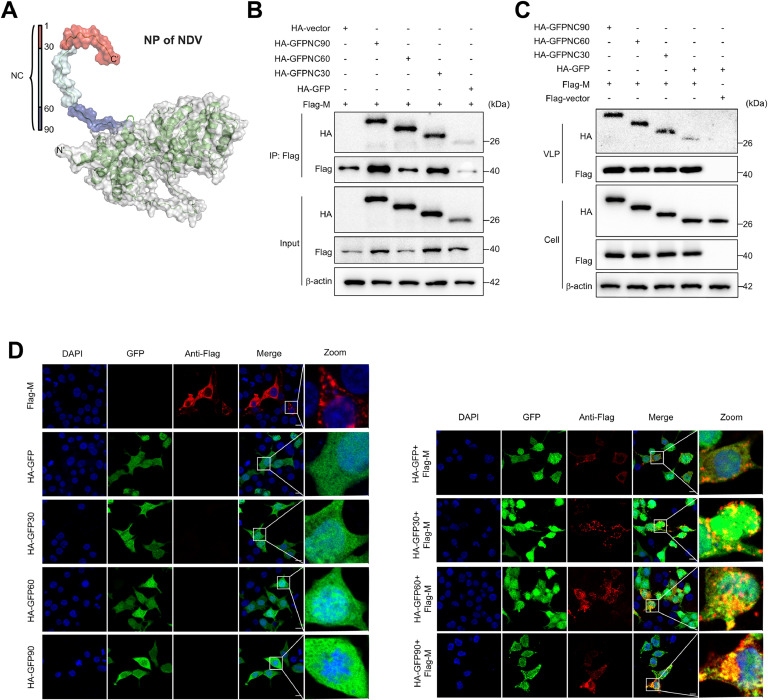
The C-terminal tail from NDV NP protein mediates the binding of GFP to NDV M proteins. (A) The three-dimensional (3D) structure of the NDV NP protein generated using the D-I-TASSER (Deep learning-based Iterative Threading ASSEmbly Refinement) modeling server (Yang et al., 2015; Zheng et al., 2025). Three segments of the C-terminus region of NDV NP protein were labeled with different colors: the last 30 amino acids (aa) in red, aa 31-60 in gray, and aa 61-90 in blue. Truncated versions of the C-terminal motifs of the NDV NP protein, were fused to the recombinant HA-GFP protein and designated as HA-GFPNC90 (90nt/30aa), HA-GFPNC60 (60nt/20aa) and HA-GFPNC30 (30nt/10aa), respectively. The interaction between the NP tail and the M protein was determined by Co-Immunoprecipitation (Co-IP) assay (B), while the ability of M protein carrying diverse NC-fused GFP proteins was determined by subsequent virus-like particle (VLP) assay (C). (D) The subcellular localization of M protein, Mock HA-GFP, and the HA-GFPNC90/60/30 constructs following co-transfection was examined by indirect immunofluorescence (IFA).

It has been reported that there is a DXD motif near the C-terminal end of Paramyxovirus NP proteins that is critical for M bringing cargo proteins into virus-like particles (VLPs) (Ray et al., 2016; Schmitt et al., 2010). As a member of the *Paramyxoviridae* family, NDV possesses a comparable DXD motif at the C-terminal end of its NP protein. The three-dimensional structure of the NDV NP protein was generated by using D-I-TASSER (Deep learning-based Iterative Threading ASSEmbly Refinement) (Yang et al., 2015; Zheng et al., 2025) in this study (Fig. 1A). It shows that the DXD motif-containing region forms a distinct "tail-like" extension at the C-terminus of NP protein, which is proposed to mediate the interaction with the M protein, as observed in other paramyxoviruses (Ray et al., 2016; Schmitt et al., 2010; Zhang et al., 2015).

To investigate the interaction between the NP tail and the M protein, truncated versions of the C-terminal motifs of the NDV NP protein, were fused to the recombinant HA-GFP protein and designated as HA-GFPNC90 (90nt/30aa), HA-GFPNC60 (60nt/20aa) and HA-GFPNC30 (30nt/10aa), respectively. Through Co-Immunoprecipitation (Co-IP) with the M protein from NDV Herts/33 strain, it was found that only the last 10 aa of the NP tail (i.e. NC30) can effectively improve the interaction with M protein, while the mock GFP could hardly interact with M protein (Fig. 1B). Correspondingly, substantial amounts of HA-GFPNC90, HA-GFPNC60, and HA-GFPNC30 proteins were detected in M-generated VLPs, suggesting that the M protein may incorporate the NC-fused GFP constructs during budding (Fig. 1C).

Indirect immunofluorescence (IFA) analysis revealed that both Mock HA-GFP and HA-GFP-NC90/60/30 constructs localized to the cytoplasm and nucleus (Fig. 1D). A partial overlap of immunofluorescence signals was observed between the M protein and the Mock HA-GFP protein, which may be indicative of co-localization. Notably, fusion of the NC90/60/30 motif significantly enhanced this cytoplasmic co-localization between GFP and the M protein, particularly near the cell membrane, suggesting that the NC motif may facilitate the recruitment of GFP into the M protein-mediated VLP budding process.

### Identification of the key amino acid residues for packaging into VLPs

**Fig. 2:**
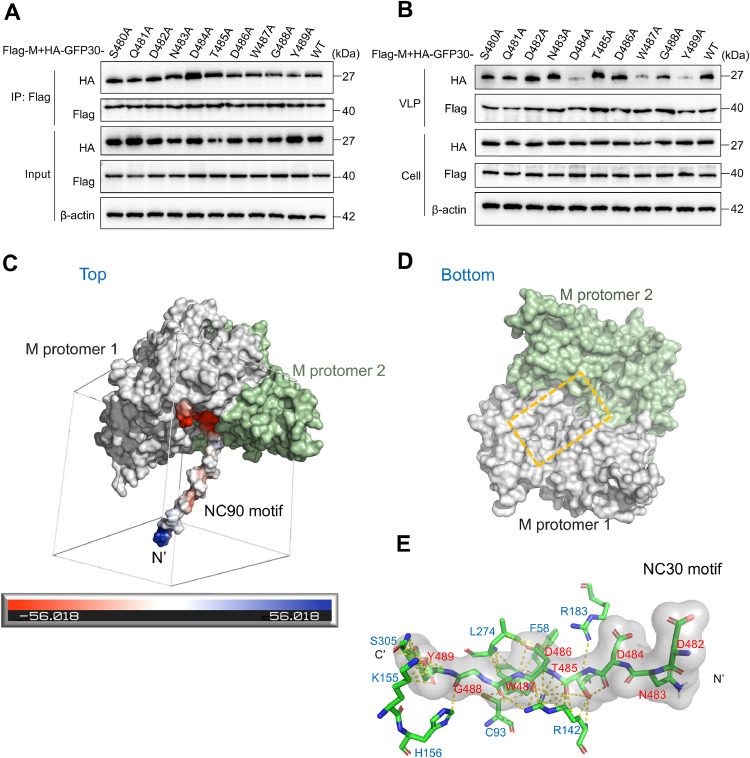
Structural model of NDV M protein and putative interaction with NP C-terminal motif. To determine the key residues within the NC30 motif, a series of site-directed mutagenesis were introduced on the eukaryotic expression plasmid of HA-GFPNC30, replacing each of the 10 amino acids with alanine one by one. (A) Co-IP analysis showed interaction between HA-GFPNC30 mutants and the M protein, while the content of the recombinant HA-GFPNC30 mutants within the M-mediated VLPs were detected by WB (B). (C) The structural model of interaction between the C-terminal motif of NDV NP protein predicted to and NDV M protein dimer (PDB code 4G1G) was generated using the ZDOCK server (Pierce et al., 2014). The two protomers of the M protein dimer are colored grey and green. Electrostatic potential at the molecular surface of NC motif were displayed, ranging from blue (56.018kT/е) to red (−56.018kT/е). There is a groove predicted at the bottom of the M protein dimer (D) and the last 8 amino acids of the NC motif (^483^NDTDNWGY^489^) could fit into it (E). Among these amino acids, the W487 and Y489 residues of the NP protein, as well as the K155, H156, C93, L274, F58, R142, and R183 residues of the M protein, play a core role in the interaction.

To determine the key residues within the NC30 motif, a series of site-directed mutagenesis were introduced on the eukaryotic expression plasmid of HA-GFPNC30, replacing each of the 10 amino acids with alanine (A) one by one. Co-IP analysis showed that the absence of these amino acid residues did not significantly affect the interaction between HA-GFPNC30 and the M protein (Fig. 2A). Nevertheless, in the subsequent VLP detection assay, it was found that the mutations D484A, W487A, and Y489A significantly reduced the content of the recombinant HA-GFPNC30 proteins in the VLPs, while the G488A mutation also had a certain reducing effect but was relatively weaker (Fig. 2B). These results indicated that the 10 amino acids at the C-terminal end of the NC motif are indeed instrumental in facilitating the entry of cargo proteins into M-yielded VLPs; however, the process is regulated by a more intricate network of mechanisms.

To delve deeper into this mechanism, the ZDOCK server (Pierce et al., 2014) was further utilized to analyze the possible interaction sites between the NP protein and the M protein dimer (Fig. 2C). It revealed that the last 7 amino acids of the NC motif (^483^NDTDNWGY^489^) could fit into the groove formed at the bottom of the M protein dimer (Fig. 2A and B). Among these amino acids, the W487 and Y489 residues of the NP protein, as well as the K155, H156, C93, L274, F58, R142, and R183 residues of the M protein, play a core role in the interaction (Fig. 2D), which perfectly aligns with the experimental results shown in Fig. 2E.

### The NC motif enhances the interaction between VP2 and NDV M proteins

**Fig. 3:**
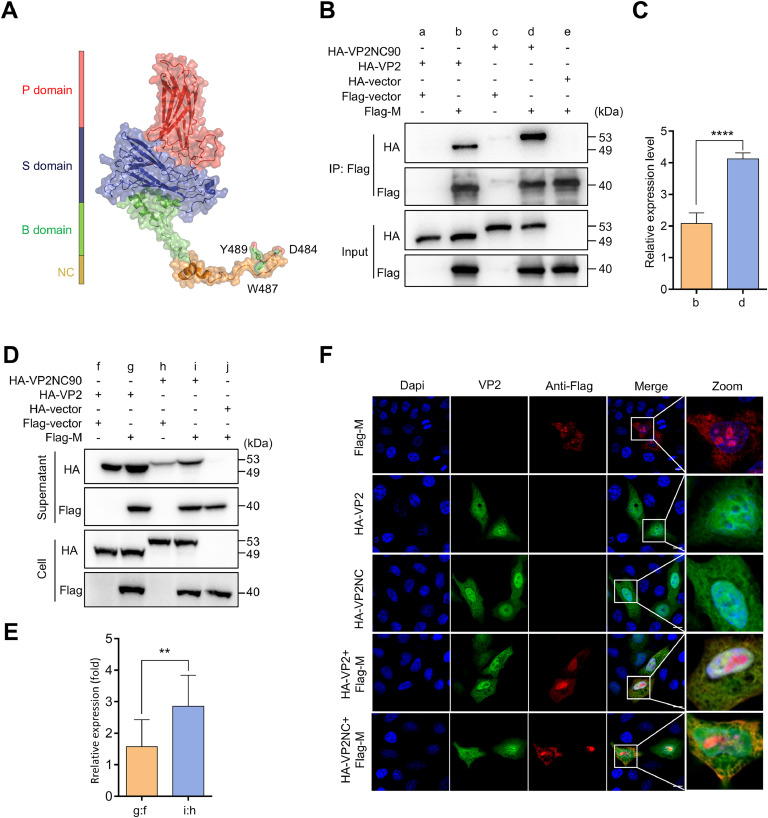
The NC motif enhances the interaction between IBDV VP2 and NDV M proteins. (A) The 3D structure of the recombinant VP2NC90 protein was predicted using D-I-TASSER (Yang et al., 2015; Zheng et al., 2025). In the modeled VP2 protein from the nVarIBDV strain SD-LY-CN-2020, the P domain, S domain, and B domain are colored red, blue, and green, respectively, and the NC90 motif is highlighted in yellow. (B) The interaction between the recombinant VP2NC protein and the M protein was determined by Co-IP assay. (C) Relative expression levels of HA-VP2NC90 and HA-VP2 were quantified and normalized to M protein based on WB band intensity. Lanes are labeled a–e above the blot. (D) The ability of M protein carrying diverse NC-fused VP2 protein was determined by the VLP assay. (E) The expression fold change of HA-VP2NC90 and HA-VP2 in the supernatant upon co-transfection with M protein, relative to transfection without M, was quantified by WB band intensity. Lanes are labeled f–j above the blot. (F) The subcellular localization of M protein, Mock HA-VP2, and the HA-VP2NC constructs following co-transfection was examined by IFA assay. Statistical significance was determined by using one-way ANOVA for multiple comparisons (* *p* < 0.05, ** *p* < 0.01, *** *p* < 0.001, **** *p* < 0.0001, *ns* = not significant).

To assess the impact of the NC motif on exogenous proteins, the NC90 motif was introduced into the C-terminus of the VP2 protein from the nVarIBDV strain SD-LY-CN-2020, thereby constructing the plasmid HA-VP2NC90. The structural prediction of the recombinant VP2NC90 protein using D-I-TASSER (Yang et al., 2015; Zheng et al., 2025) indicates that the VP2 part of the recombinant protein maintains its native structure, whereas the NC90 motif part will project outward as a tail (Fig. 3A).

The Co-IP assay indicates that, despite originating from different viral sources, there is a direct or indirect interaction between the VP2 and M proteins (Fig. 3B). Notably, fusing the NC90 motif to the VP2 protein leads to a significant enhancement in its binding affinity to the M protein, and this enhancement is statistically significant (*p* < 0.0001) (Figs. 3C). This enhanced interaction was further confirmed in the VLPs assay (Figs. 3D and 3E). It is well known that both the VP2 and M proteins are capable of forming VLPs, which are released into the cell supernatant. Co-transfection with a plasmid expressing the M protein results in a slight increase in the VP2 protein content within the VLPs purified from the supernatant. In contrast, the co-expression of the M protein significantly increases the concentration of VP2NC90 in the supernatant, with a statistically significant difference (*p* < 0.01) (Fig. 3E). Subsequent IFA assay demonstrates that the fusion of the NC90 motif significantly increases the colocalization of VP2 and M proteins at the cell membrane (Fig. 3F). This finding further confirms that the NC90 motif can play a role in promoting VP2′s involvement in the M-mediated budding process.

### Biological characteristics of recombinant NDV strains expressing nVarIBDV VP2

**Fig. 4:**
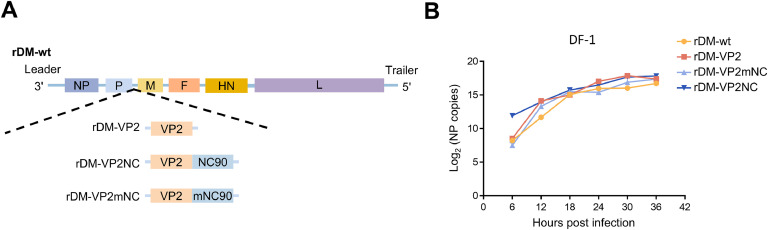
Impact of foreign VP2 gene insertion on the replication of recombinant NDV strains. (A) Schematic of recombinant NDV strains. The parental control rLaSota-DM wild type strain (rDM-wt), containing R247K and S263R mutations in the M protein (Peng et al., 2022), served as the backbone. Expression cassettes for the nVarIBDV VP2 protein, as well as the VP2NC90 and VP2mNC90 fusion proteins, were individually inserted between the P and M genes. In VP2NC90, VP2 is fused to the NP-derived NC motif from the LaSota strain, whereas VP2mNC90 contains a codon-optimized version of this motif to avoid potential interference caused by sequence identity with the LaSota vector backbone. (B) Multistep growth kinetics. DF1 cells were infected in triplicate with rDM-wt, rDM-VP2, rDM-VP2NC90, or rDM-VP2mNC90 at an MOI of 5. To assess replication kinetics, culture supernatants were harvested every 6 hours post-inoculation, and virus titers were determined by RT-qPCR.

The findings from prior research suggest that the NC motif can strengthen the binding affinity between the exogenous protein VP2 and the M protein. This leads to the hypothesis that incorporating a fused NC motif into VP2 within the NDV vector could potentially enhance its integration within viral particles. To test this hypothesis, four distinct recombinant viruses were generated. As illustrated in Fig. 4A, the rLaSota-DM wild type strain, referred to as rDM-wt, serves as the parental control (DM stands for double mutation and refers to the K247R and S263R mutation of the M protein). This strain was previously constructed by introducing R247K and S263R mutations into the M protein of the LaSota strain and has been validated as a promising vaccine candidate, having been optimized for virus budding efficiency and titers (Peng et al., 2022). Based on the rDM genome, the expression cassette for the nVarIBDV VP2 protein gene, as well as those for the VP2NC90 and VP2mNC90 fusion proteins, were inserted between the P gene and the M gene independently. In the VP2NC90 construct, the VP2 protein is fused with the NP NC motif derived from the LaSota strain, whereas the VP2mNC90 construct employs a codon-optimized NC motif.

The recombinant viruses were passaged in chicken embryos, and the fifth-generation viruses were used for subsequent biological and immunological analysis. As shown in Table 2, there is no significant difference in the HI titers among the four recombinant viruses. Nevertheless, the EID_50_ values for the rDM-VP2NC90 and rDM-VPmNC90 strains were higher than those of rDM-VP2, and were comparable to those of the parental strain rDM. The kinetics and final virus yield were determined under multistep growth conditions on DF1 cells. Each recombinant virus — rDM, rDM-VP2, -VP2NC90, and -VP2mNC90 — was inoculated onto triplicate layers of DF1 cells at a multiplicity of infection (MOI) of 0.01. Samples were harvested every 6 hours to assess the virus titers using reverse transcription quantitative polymerase chain reaction (RT-qPCR) assay. The results showed that the kinetics and magnitude of replication for four NDV strains were very similar, and no significant differences were observed (Fig. 4B).

**Table 2:** Results for fifth generation of recombinant viruses.

Virus	Log_2_HA		lgEID_50_^b^	MDT^c^	ICPI^d^
	Mean±SEM^a^	Max			
rDM-wt	11.60 ± 0.88	13	−9.22	102 h	0
rDM-VP2	11.53 ± 0.81	12	−8.83	127.2 h	0
rDM-VP2NC	11.67 ± 0.87	13	−9.17	130.4 h	0
rDM-VP2mNC	11.73 ± 0.68	13	−9.17	133 h	0

a SEM, standard error of the mean.

b lgEID_50_, logarithm base 10 of 50 % egg culture infective dose.

c MDT, mean death time.

d ICPI, intracerebral pathogenicity index.

The virulence of the parental virus rDM and the recombinant viruses rDM-VP2, rDM-VP2NC90, and rDM-VP2mNC90 were determined using the intracerebral pathogenicity index (ICPI) and mean death time (MDT) assays. All four recombinant NDV strains exhibited an ICPI value of 0 and MDT values greater than 90 hours, which classified them into the lentogenic group. Notably, the recombinant strains rDM-VP2, rDM-VP2NC90, and rDM-VP2mNC90 displayed slightly higher MDT values than the parental rDM strain, suggesting that the pathogenicity of the recombinant viruses was not increased after the insertion of the nVarIBDV VP2 gene.

### The NC motif effectively enhances the abundance of VP2 protein in recombinant viruses

**Fig. 5:**
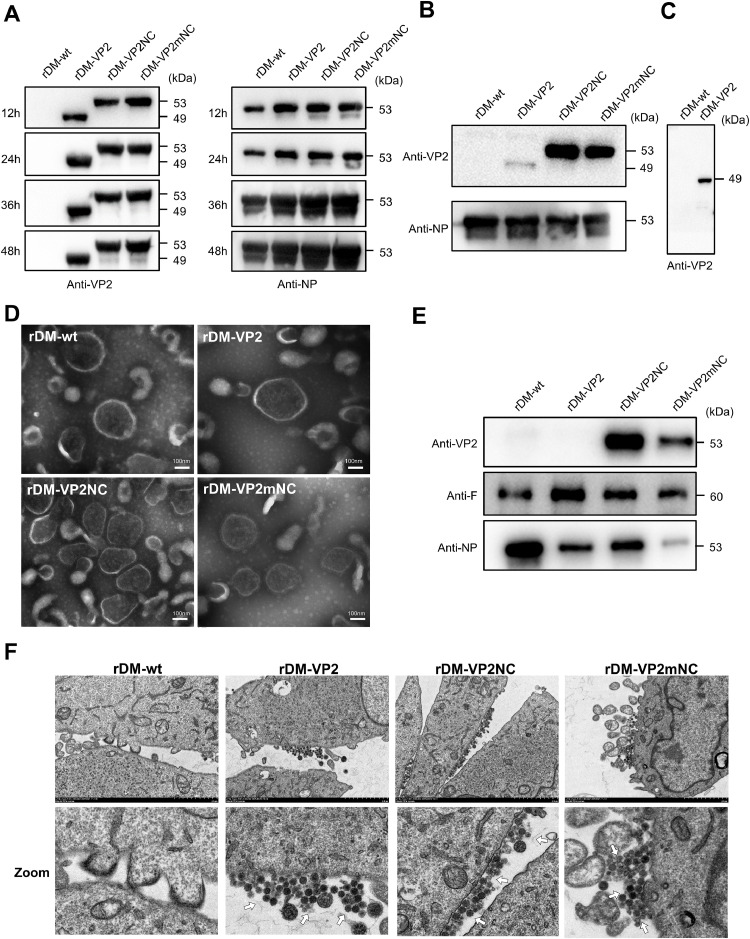
The NC motif enhances VP2 protein abundance in recombinant NDV strains. (A) Viral protein expression in infected cells. DF1 cells were infected with rDM-VP2, rDM-VP2NC90, or rDM-VP2mNC90 at an MOI of 5. Expression levels of the viral NP protein and the inserted recombinant VP2 proteins were analyzed by Western blot. (B) VP2 protein in allantoic fluid. The content of recombinant VP2 protein was assessed by Western blot in fresh allantoic fluid harvested from SPF embryonated chicken eggs inoculated with the indicated recombinant viruses. (C) Extended exposure of rDM‑VP2 allantoic fluid. The low content of VP2 protein in the rDM‑VP2 allantoic fluid was further examined using an extended exposure time. (D) Morphology of purified virus particles. The four recombinant viruses were purified by ultracentrifugation and visualized by transmission electron microscopy (TEM). Scale bars represent 100 nm. (E) VP2 content in purified virions. The relative amount of recombinant VP2 protein incorporated into purified viral particles of rDM‑wt, rDM‑VP2, and rDM‑VP2NC90 was detected by Western blot. (F) TEM of infected cells. Cells infected with the four recombinant viruses were visualized by TEM.

Subsequently, the expression of viral proteins of the recombinant NDV strains rDM-VP2, rDM-VP2NC90, and rDM-VP2mNC90 were examined in cell cultures. After infecting DF1 cells with the same dose of 5 MOI, WB assay showed that the expression levels of NP protein and VP2 protein in infected cells were essentially identical among the four recombinant virus strains, with no significant differences observed (Fig. 5A). However, when WB analysis was performed on fresh allantoic fluid containing each of the four recombinant viruses, it was found that the expression levels of VP2 protein in rDM-VP2NC90, and rDM-VP2mNC90 were significantly higher than those in the traditionally constructed recombinant virus rDM-VP2, with extremely notable differences (Fig. 5B). Despite the extended exposure time in Fig. 5C indicating that exogenous VP2 protein could be effectively expressed in rDM-VP2, the amount of VP2 in rDM-VP2 was nearly undetectable when compared to the levels in rDM-VP2NC90 and rDM-VP2mNC90 in Fig. 5B.

To further verify the role of the NC motif in the incorporation of exogenous proteins into virus particles, we proceeded to isolate and purify the four recombinant viruses via ultracentrifugation. Transmission electron microscopy (TEM) observation of the purified virus particles showed no significant differences in morphology and particle diameter between the three recombinant viruses expressing exogenous VP2 proteins and the parental strain (Fig. 5D). No suspected IBDV VLPs were found inside recombinant NDV virions. Further WB analysis revealed that substantial amounts of VP2 protein were detected in the purified virions of rDM-VP2NC90 and rDM-VP2mNC90, whereas no VP2 protein was detected in rDM-VP2 virions, similar to the parental strain (Fig. 5E). These results suggest that foreign VP2 proteins cannot be incorporated into NDV virions without the assistance of the NC motif, and that the NC motif indeed confers the ability to carry VP2 protein into NDV virions.

To further characterize the form of VP2 protein associated with NDV virions, virus‑infected cells were examined by electron microscopy. Large numbers of IBDV VLPs expressed by rDM‑VP2, rDM‑VP2NC, and rDM‑VP2mNC were observed predominantly in the extracellular space (Fig. 5F), with only sporadic VLPs detected intracellularly (data not shown). In contrast, few suspected IBDV VLPs were found inside recombinant NDV virions (Fig. 5F), which is consistent with the electron microscopy observation of purified virions, indicating that IBDV VP2 is not incorporated into recombinant NDV virions in the form of VLPs. Notably, cells infected with rDM‑VP2NC or rDM‑VP2mNC displayed more extensive regions of VLP accumulation compared with rDM‑VP2‑infected cells, suggesting that the NC motif may promote VLP formation and release (data not shown).

**Fig. 6:**
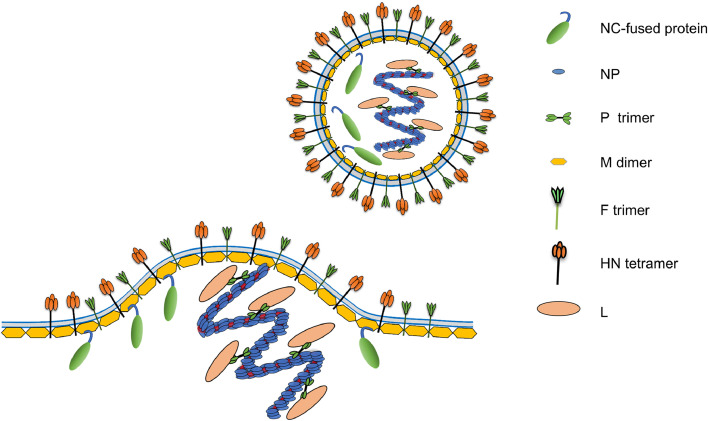
Schematic model of NC-mediated incorporation of foreign VP2 into viral particles. The illustration proposes a mechanism in which the NC motif facilitates the packaging of foreign VP2 into budding virions, likely by mimicking the recruitment pathway of the viral ribonucleoprotein (RNP) complex.

Collectively, these results imply that NC can mediate the incorporation of foreign VP2 proteins into virus particles probably through a mechanism similar to that of the RNP complex (see schematic diagram shown in Fig. 6).

### The NC motif significantly improves the protective efficacy of the recombinant NDV/VP2 against nVarIBDV challenge

**Fig. 7:**
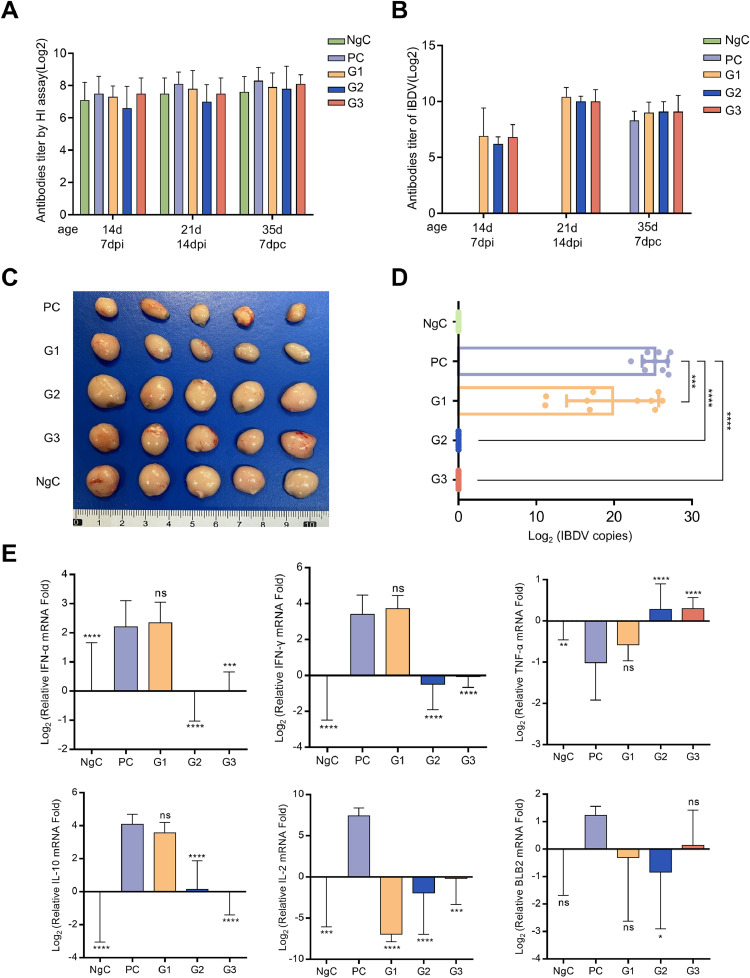
The NC motif enhances the protective efficacy of recombinant NDV-vectored VP2 vaccines against nVarIBDV challenge. Groups of 7-day-old chickens were immunized via the intraocular and nasal routes with rDM-wt (wild-type control), rDM-VP2, rDM-VP2NC90, or rDM-VP2mNC90. At 21 days post-immunization, birds were challenged with 50 BAD₅₀ of the nVarIBDV strain SD-LY-CN-2020. (A) NDV- and (B) IBDV-specific antibody titers at 7 and 14 dpi, and 7 days post-challenge (dpc). At each time point, there were no statistically significant differences in the values among the PC, rDM-VP2, rDM-VP2NC90, and rDM-VP2mNC90 groups. (C) Representative images of bursae from vaccinated and control groups at 21 days post-infection (dpi). IBDV viral load (D) and relative expression levels of immune‑related factors in the bursa of Fabricius (E) were measured by qPCR. The values of each group were statistically compared with those of the PC group. Statistical significance was determined by using one-way ANOVA for multiple comparisons (* *p* < 0.05, ** *p* < 0.01, *** *p* < 0.001, **** *p* < 0.0001, *ns* = not significant).

To determine the immunogenicity and protective efficacy of the recombinant NDV strains expressing VP2 proteins against nVarIBDV, 5 groups of 7-day old chickens were vaccinated intraocularly and nasally with rDM-wt, rDM-VP2, rDM-VP2NC90, or rDM-VP2mNC90 as shown in Table 3. One and two weeks after vaccination, blood samples were detected by Hemagglutination Inhibition (HI) and Enzyme-Linked Immunosorbent Assay (ELISA) for the titers of antibodies against NDV or IBDV. Our results showed that there is no obvious difference of antibody titers between chickens immunized with those NDV strains (Fig. 7A and 7B), suggesting each of the VP2, VP2NC or VP2mNC proteins expressed by recombinant NDV (rNDV) strains induced a very good immune response in chickens and the expression of either VP2 or VP2NC did not significantly influence the immunogenicity of NDV.

**Table 3:** Animal experimental design for SPF chickens against nVarIBDV.

Group	Immunization				Challenge			
	Strain	Dose (EID_50_)	age	Route	Strain	Dose (BAD_50_)	age	Route
NgC^a^	rDM-wt	10^7^	7d	I.N.^c^	PBS	/	28d	I.N.
PC^b^	rDM-wt	10^7^	7d	I.N.^c^	SD-LY-CN-2020	50	28d	I.N.
G1	rDM-VP2	10^7^	7d	I.N.	SD-LY-CN-2020	50	28d	I.N.
G2	rDM-VP2NC	10^7^	7d	I.N.	SD-LY-CN-2020	50	28d	I.N.
G3	rDM-VP2mNC	10^7^	7d	I.N.	SD-LY-CN-2020	50	28d	I.N.

a NgC: Negative control for nVarIBDV challenge.

b PC: Positive control for nVarIBDV challenge.

c I.N.: intraocular-nasal vaccination.

At 21 days post-immunization, immunized chickens and unvaccinated positive controls were challenged with Bursa-cultured nVarIBDV strain SD-LY-CN-2020 at a dose of 50 BAD₅₀ (equivalent to approximately 6 × 10^6^ viral RNA copies) in 100 μL PBS per bird (Table 3). Consistent with previous studies (Fan et al., 2019b; Huang et al., 2022; Takahashi et al., 2024; Zhang et al., 2026), no overt clinical signs were observed in the positive control chickens after nVarIBDV challenge; however, marked bursal atrophy was evident (Fig. 7C), resulting in a 100 % incidence rate (Table 4).

**Table 4:** Protection efficacy for SPF chickens against nVarIBDV after vaccination.

Group	Strain	Challenge strain	Bursa/Weight^a^	BBIX^b^	Incidence (%)^d^	PI (%)^e^
			Mean±SEM^c^			
NgC	rDM-wt	PBS	3.70 ± 0.78	/	/	/
PC	rDM-wt	SD-LY-CN-2020	1.28 ± 0.29	0.34 ± 0.08	100	/
G1	rDM-VP2	SD-LY-CN-2020	2.41 ± 0.53	0.65 ± 0.14	80	20
G2	rDM-VP2NC	SD-LY-CN-2020	4.38 ± 0.94	1.18 ± 0.25	0	100
G3	rDM-VP2mNC	SD-LY-CN-2020	4.51 ± 0.35	1.22 ± 0.10	0	100

a Bursa/Weight, the ratio of bursa to body weight.

b BBIX, bursa: body-weight index. BBIX$=\frac{\text{bursa}/\text{body}\;\text{weight}\;\text{ratio}\;\text{of}\;\text{challenged}\;\text{group}}{\text{bursa}/\text{body}\;\text{weight}\;\text{ratio}\;\text{of}\;\text{unchallenged}\;\text{control}\;\text{group}}$ . The criteria for bursal atrophy were determined according to conventional standards, where a BBIX value of less than 0.7 was considered indicative of bursal atrophy (Fan et al., 2020; Lucio and Hitchner, 1979).

c SEM, standard error of the mean.

d Incidence is defined as the percentage of experimental chickens exhibiting any of the following: disease symptoms, bursal atrophy, or death. Incidence (%) = Percentage of the sick or dead chickens until 7 days post-challenge.

e PI = Protective index. $\text{PI}\left(\%\right)=\frac{\text{Incidence}\;\text{of}\;\text{challenge}\;\text{control}\;-\;\text{Incidence}\;\text{of}\;\text{each}\;\text{experimental}\;\text{group}}{\text{Incidence}\;\text{of}\;\text{challenge}\;\text{control}}\times 100\%$.

**Fig. 8:**
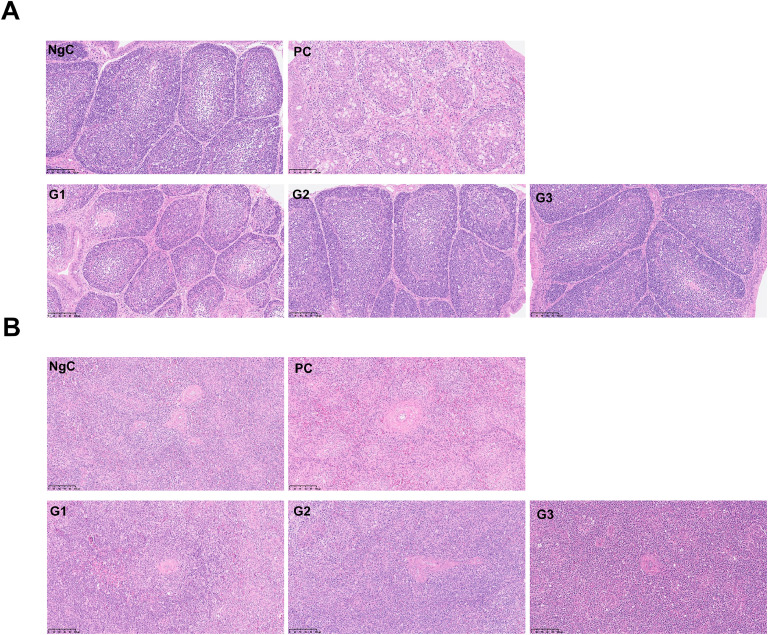
Histopathological assessment of bursa and spleen after nVarIBDV challenge. Representative hematoxylin and eosin (H&E) staining images of the bursa of Fabricius (A) and spleens (B) from the indicated groups at 7 days post-challenge (dpc) are shown for each group.

The three immunization groups exhibited varying levels of protection. The bursae from the rDM-VP2NC and rDM-VP2mNC groups showed no noticeable size differences compared to the healthy control group following challenge (Fig. 7C). Viral RNA was barely detectable in chickens immunized with rDM-VP2NC or rDM-VP2mNC. In contrast, immunization with rDM-VP2 significantly reduced, but did not eliminate, the viral load in the bursa of Fabricius (Fig. 7D). The bursa-to-body weight index (BBIX) values for both the rDM-VP2NC and rDM-VP2mNC groups exceeded 1, confirming the absence of bursal atrophy (Table 4). Histopathological examination via HE staining further demonstrated that rDM-VP2NC and rDM-VP2mNC effectively protected of the bursa of Fabricius (Fig. 8A), and no obvious lesions were observed in the spleens of any group (Fig. 8B). In the bursa of Fabricius of challenge control group, severe follicular wrinkling, connective tissue hyperplasia, and medullary depletion due to inflammatory damage were observed. In contrast, bursae from the rDM-VP2NC and rDM-VP2mNC groups retained normal architecture, with regular follicle size and spacing, and well-preserved medullary structure. In comparison, the conventionally constructed rDM-VP2 strain provided inferior protection. Bursae in this group were significantly smaller than those in the healthy controls, with a mean BBIX of only 0.65 ± 0.14. Pathological analysis revealed intact but somewhat reduced in size, indistinct connective tissue borders at the follicular margins, and pallor of the cortical regions.

We further examined the relative expression levels of immune‑related factors in the bursa of Fabricius of experimental chickens at 7 dpc (Fig. 7E). In the challenge control group, the expression levels of IFN‑α, IFN‑γ, IL‑10, and IL‑2 were all significantly upregulated, indicating persistent infection and ongoing inflammatory responses. In contrast, the group immunized with rDM‑VP2, which was constructed using the conventional approach, also showed significant upregulation of IFN‑α, IFN‑γ, and IL‑10, but IL‑2 was significantly downregulated. This pattern suggests that the conventional vaccine failed to effectively clear the virus or suppress inflammation and may be associated with impaired T‑cell immune responses. For the rDM‑VP2NC and rDM‑VP2mNC groups, the expression levels of all four cytokines were not significantly different from those of the healthy control group, or showed a slight downregulation. Taken together with the viral load (Fig. 7D) and histopathological observations (Fig. 8A), these results imply that chickens in the NC‑fused groups successfully cleared the challenge virus, resolved inflammation, and returned to an immune quiescent/homeostatic state.

Overall, rDM-VP2NC and rDM-VP2mNC provided stronger protection against nVarIBDV challenge than did rDM-VP2, suggesting that the NC motif can enhance the immunogenic efficacy of exogenous proteins delivered by an NDV vector.

## Discussion

In this study, a novel strategy was developed to enhance the incorporation of foreign proteins into NDV virions by leveraging the virus's intrinsic assembly system. The effects of this strategy on the biological properties and immunogenicity of the recombinant virus have been evaluated. Previous evidence has suggested that a higher incorporation efficiency of foreign proteins in vector viruses can boost their immunogenicity (Buonocore et al., 2002; Kretzschmar et al., 1997; Li et al., 2021; Malherbe et al., 2021; Sun et al., 2020). An established method involves exchanging the transmembrane and cytoplasmic domains of foreign glycoproteins with those of the vector virus to exploit its assembly machinery. Here, we extend this principle to non-membrane proteins by utilizing the vRNP complex assembly pathway of NDV to increase their incorporation into virions.

During paramyxovirus assembly, the vRNP is incorporated into the virion through interactions between the M protein and the N protein (where N of other paramyxoviruses corresponds to NDV's NP, differing only in nomenclature) (El Najjar et al., 2014; Ray et al., 2016). The M protein initially accumulates locally at the inner side of the cell membrane and forms a lattice structure through intermolecular interactions (El Najjar et al., 2014), thereby establishing the budding zone (Battisti et al., 2012; Bringolf et al., 2017) (Fig. 6). With its negatively charged surface, the vRNP binds to the basic residue-rich M protein, facilitating its entry into the budding zone (Watkinson and Lee, 2016; Zhang et al., 2014). Meanwhile, the M protein interacts with the transmembrane domains of glycoproteins F and HN (El Najjar et al., 2014), coordinating the integration of these viral components into the budding vesicle and completing the budding process (El Najjar et al., 2014; Watkinson and Lee, 2016; Zhang et al., 2014).

Studies on paramyxoviruses such as parainfluenza virus, mumps virus, and Nipah virus have demonstrated the critical role of the N protein's C-terminal region in M protein-mediated budding. Point mutations in the C-terminus of N can disrupt NP-M interactions and impair VLP formation (Ray et al., 2016; Schmitt et al., 2010). Furthermore, it is reported that the conserved C-terminal DXD motif in paramyxovirus N proteins enables the incorporation of foreign proteins into VLPs (Ray et al., 2016). Ray et al. (2016) predicted that the DXD motif of the NDV NP protein is likely located within ^483^NDTD^486^, although this has not been experimentally validated (Ray et al., 2016).

In this study, we functionally identified, for the first time, the C-terminal NC motif of the NDV NP protein and the potential DXD motif. Functional assays demonstrated that the last ten residues (481–489) of the C terminus of the NP protein mediate the incorporation of foreign proteins into both VLPs and virions through interaction with the M protein (Fig. 1, Fig. 2). Structural modeling further indicated that the last eight residues (^483^NDTDNWGY^489^) dock into the groove at the bottom of the M protein dimer (Figs. 2C and 2D), supporting direct physical interaction. In VLP incorporation assays, three mutants—D484A, W487A, and Y489A—significantly impaired the incorporation of foreign proteins (Fig. 1D). While D484 lies within the previously predicted DXD motif, W487 and Y489 are located outside of the motif, suggesting that the NC motif functions through a cooperative, multi-residue mechanism, rather than solely depending on the DXD sequence. These findings extend beyond the DXD model proposed by Ray et al. (Ray et al., 2016), indicating that additional residues in the NP C-terminus contribute to M-mediated budding and incorporation. This study provides preliminary evidence supporting the feasibility of utilizing the NDV NC motif to mimic the viral assembly process and mediate the entry of foreign proteins into virions, thereby establishing an experimental basis for further applied research.

To evaluate the utility of the NC motif, a series of recombinant NDV strains that stably expresses the nVarIBDV VP2 protein were employed in this study. The VP2 protein is well-established as the major protective antigen against IBDV, with numerous studies demonstrating its good immunogenicity (Azad et al., 1991; Becht et al., 1988; Heine and Boyle, 1993; Huang et al., 2004; Liu et al., 2024). It has been successfully incorporated into multiple genetically engineered vaccine platforms, including nucleic acid vaccines (Hsieh et al., 2010; Mahmood et al., 2006), VLP vaccines (Li et al., 2020; Wang et al., 2021b), and virus-vectored vaccines based on NDV (Dey et al., 2017; Huang et al., 2004), fowl pox virus (FPV) (Boursnell et al., 1990), turkey herpesvirus (HVT) (Shah et al., 2022), and Marek's disease virus (MDV) (Tsukamoto et al., 1999). Given this evidence, VP2 is considered a key antigen for the development of genetically engineered vaccines against nVarIBDV in this study.

To facilitate efficient incorporation of VP2 into NDV virions, the C-terminal 30 amino acids of the NP protein (designated as NC90) were selected as a fusion peptide. This design aims to assess the effect of NC90 on recombinant protein expression and immunogenicity while preserving the structural and functional integrity of the NC motif. Structural analysis of the NDV NP protein reveals that the NC90 region is entirely exposed outside the NP core, whereas the minimal NC30 corresponds only to the extreme C‑terminal tail. Although NC30 retains the core binding motif, the additional residues in NC90 may function as a flexible linker, ensuring proper spatial orientation and accessibility of the NP protein when interacting with the virion‑associated M protein during virus assembly (Kho et al., 2003; Nath et al., 2020; Ray et al., 2016). Therefore, in this design, the more extended NC90, which closely mimics the native conformation of the NP C‑terminal domain, is expected to support more reliable and efficient incorporation of VP2 into NDV virions.

We successfully rescued four recombinant NDV strains: the parental rDM-wt and three derived strains—rDM-VP2, rDM-VP2NC90, and rDM-VP2mNC90. In the constructs rDM-VP2NC90 and rDM-VP2mNC90, the same amino acid sequence of the NC90 motif was used, although their nucleotide sequences differ. Specifically, rDM-VP2NC90 uses the original codon sequence of the LaSota strain, whereas rDM-VP2mNC90 employs a more common codon sequence to avoid potential interference caused by sequence identity with the LaSota vector backbone. No significant differences were observed among these strains in ICPI value, HI titer in embryonated eggs, or replication efficiency in cells. However, rDM-VP2, rDM-VP2NC90, and rDM-VP2mNC90 exhibited slightly higher MDT values than rDM-wt, indicating that the insertion of a foreign gene moderately attenuated viral virulence—a phenomenon commonly reported in related studies (Ge et al., 2011; Hu et al., 2020; Huang et al., 2004; Peng et al., 2022). Notably, the three foreign gene-inserted strains shared highly similar biological characteristics and virulence levels, suggesting that the introduction of either the wild-type or codon-optimized NC motif did not significantly alter the replication or pathogenicity of the recombinant viruses.

The recombinant VP2 proteins expressed by rDM-VP2, rDM-VP2NC, and rDM-VP2mNC were systematically evaluated. Generally, foreign proteins expressed by recombinant viral vectors cannot be incorporated into virions and are produced only inside infected cells. To overcome this limitation, we introduced the NC motif and systematically evaluated its ability to facilitate foreign protein incorporation into NDV virions. As a control, the rDM-VP2 strain was constructed according to the conventional design (Dey et al., 2017; Huang et al., 2004; Liu et al., 2024). First, in infected cells, all three recombinant viruses (rDM-VP2, rDM-VP2NC, and rDM-VP2mNC) efficiently expressed comparable levels of VP2 protein. In contrast, in virus-inoculated chicken embryos, the amounts of VP2 protein released into the allantoic fluid were significantly higher for rDM-VP2NC and rDM-VP2mNC than for rDM-VP2, which is consistent with our experimental design. Subsequently, after purifying NDV virions by ultracentrifugation, we found that the purified product of rDM-VP2 contained no VP2 protein, whereas those of rDM-VP2NC and rDM-VP2mNC were rich in VP2 protein, indicating that the NC motif indeed confers the ability to carry VP2 protein into NDV virions. To further characterize the form of VP2 protein incorporated into NDV virions, infected cells were examined by electron microscopy. The results revealed that large amounts of IBDV VLPs expressed by rDM-VP2, rDM-VP2NC, and rDM-VP2mNC were released into extracellular space. However, suspected IBDV VLPs were rarely observed within recombinant NDV virions, suggesting that VP2 is incorporated into rDM-VP2NC and rDM-VP2mNC virions not as VLPs, but rather in monomeric or multimeric form.

In terms of immunogenicity, each virus effectively elicited humoral immune responses of similar magnitude, indicating that the NC motif does not compromise the immunogenicity of VP2 or the NDV vector. Notably, although rDM-VP2NC and rDM-VP2mNC induced humoral immunity comparable to that of rDM-VP2, both conferred stronger overall protections. This enhanced protective efficacy was evidenced by bursal size and BBIX indices similar to those of the healthy control group, along with the absence of pathological lesions in bursal tissue. As shown in Fig. 7E, we examined the expression of cytokines in the bursa of Fabricius of the experimental chickens. The results showed that on day 7 post-challenge, the expression levels of most cytokines were downregulated or unchanged in chickens immunized with rDM-VP2NC and rDM-VP2mNC. Taken together with the “no virus, no lesion” status observed in these chickens based on viral load (Figs. 7E) and histopathological analyses (Figs. 8A), this indicates that the observed phenomenon is not immunosuppression but rather a hallmark of the immune system having efficiently cleared the virus and rapidly returned to a resting state. The complete viral clearance in the bursa of Fabricius achieved by rDM-VP2NC and rDM-VP2mNC may be a key reason for their significantly better protective efficacy compared with rDM-VP2.

Our studies have shown that VP2 is incorporated into rDM-VP2NC and rDM-VP2mNC virions but not into rDM-VP2 virions. Considering that NDV is a virus with broad infectivity capable of spreading to multiple tissues and organs, the recombinant NDV virions carrying VP2 protein distribute VP2 to a wider range of tissues, making it more readily recognized by the immune system and thereby eliciting a broader immune response. This may explain why chickens immunized with rDM-VP2NC and rDM-VP2mNC achieve more efficient clearance of the challenge IBDV and exhibit higher protective efficacy. Based on these findings, it is reasonable to speculate that the high level of VP2 incorporation into virions would likely enhance its recognition and presentation by the host immune system, thereby effectively enhancing the immunogenicity of the recombinant virus. Furthermore, using this NC fusion technology, anti‑tumor factors could be fused with the NC motif, packaged into virions, and released upon viral entry, potentially synergizing immune modulation and oncolysis. Nevertheless, the underlying molecular mechanisms and methodologies remain to be further explored.

In conclusion, we engineered the rDM-VP2NC and rDM-VP2mNC strains by leveraging the NDV vRNP assembly pathway. These strains provided stronger protection against nVarIBDV than rDM-VP2, supporting the role of the NC motif in enhancing the expression and immunogenicity of NDV-delivered antigens. Moreover, rDM-VP2NC led to significantly higher foreign protein incorporation into virions, highlighting a strategy to establish NDV vectors that directly deliver peptide- or protein-based therapeutics without relying on viral replication. This fusion strategy for foreign proteins offers valuable insights for vaccine development and oncolytic virotherapy.

## Authors’ contribution

CD and XQ conceived and designed the experiments. ZZ, CL, YZ, and JD performed the experiments, optimized protocols, and acquired data. LT, CS, NT, YL, YS, YQ, and CD analyzed and interpreted the data and contributed to analytical tools. ZZ and XQ drafted the manuscript. All authors critically revised the manuscript for important intellectual content, approved the final version, and agree to be accountable for all aspects of the work.

## Consent for publication

All authors consented to publish this manuscript.

## Disclosures

We declare that we have no known competing financial interests or personal relationships that could have appeared to influence the work reported in this manuscript entitled “Engineered VP2-NC antigen expressed in an NDV vector elicits enhanced protection against the challenge of the novel variant Infectious Bursal Disease Virus”.
